# Hereditary transthyretin amyloidosis in mainland China: a unicentric retrospective study

**DOI:** 10.1002/acn3.51328

**Published:** 2021-03-19

**Authors:** Kang Du, Fan Li, Hui Wang, Yuanfeng Miao, He Lv, Wei Zhang, Zhaoxia Wang, Yun Yuan, Lingchao Meng

**Affiliations:** ^1^ Department of Neurology Peking University First Hospital 8 Xishiku Street Xicheng District Beijing 100034 China

## Abstract

**Objective:**

This study aims to report the genotypes and phenotypes of hereditary transthyretin amyloidosis **(**ATTR) in a large Chinese cohort, yet the clinical and genetic profiles of ATTR remain elusive in mainland China.

**Methods:**

Fifty‐four patients with molecularly confirmed ATTR from 39 unrelated families were identified by sequencing the *TTR* gene. Sural nerve biopsies were performed in 40 of these cases. The clinical and electrophysiological data were retrospectively collected and analyzed.

**Results:**

The male/female ratio was 42:12. The average age of patients at the onset of the disease was 47.8 ± 13.0 years. The late‐onset type occurred in 29 cases (53.7%). Twenty‐two probands (56.4%) had a family history with ATTR. The initial symptoms were limb paresthesia in 33 cases (61.1%), autonomic dysfunction in 15 cases (27.8%), and blurred vision in 6 cases (11.1%). A total of 22 different *TTR* mutations were identified, including Val30Met (25.6%) in 10 families in North China and Ala97Ser in 4 families (10.3%) in South China. Electrophysiological studies revealed general sensorimotor axonal polyneuropathy in 33/44 cases (75.0%), mixed neuropathy with axonal and demyelinating impairment features in 9/44 cases (20.5%) and isolated carpal tunnel syndrome in two cases. Sural nerve biopsies revealed positive Congo red staining in 16/40 cases (40.0%).

**Conclusion:**

Chinese patients with ATTR exhibited heterogeneous *TTR* genotypes and clinical phenotypes. Val30Met remains the most common mutation type in mainland China.

## Introduction

Hereditary transthyretin amyloidosis (ATTR) is a progressive and life‐threatening disease caused by *TTR* gene mutation with an autosomal dominant inheritance pattern. The disease is characterized by TTR amyloid fibrils deposition in multiple organs, leading to sensory‐motor neuropathy, autonomic dysfunction, and cardiomyopathy, accompanied by vitreous opacities and renal insufficiency.^1^ According to the age of onset, ATTR is classified as early‐onset before 50 years old and late‐onset after 50 years old. Slowly progressive polyneuropathy and cardiomyopathy are more common in the late‐onset patients, while rapidly progressive polyneuropathy with prominent autonomic dysfunction is common in the early‐onset ones.^2^ Electrophysiological studies are generally compatible with sensory‐motor axonal polyneuropathy, but sometimes with demyelinating features.^3^ Pathologically, the loss of unmyelinated fibers occurs early, reduced density of small and then larger myelinated fibers is observed, and blood vessels are frequently invaded and destroyed by TTR accumulation.^4^ Diagnosis of ATTR mainly depends on amyloid deposits in the tissues and *TTR* gene testing. So far, more than 150 mutations have been described in the *TTR* gene.^5^ The prevalence of different mutations varies according to ethnicity and geographic locations. *TTR* Val30Met mutation is the most common pathogenic variant overall.^4^

ATTR is a phenotypically and geographically variable disease.^6^ There is a great phenotypic variability between patients, but length‐dependent axonal, sensory‐motor and autonomic polyneuropathies with varying degrees of other organ involvement are the distinct features of ATTR.^7^, ^8^ In Taiwan, *TTR* Ala97Ser is the most common mutation.^9^, ^10^ However, a limited number of cases of ATTR have been reported in the form of case reports in mainland China.^11^, ^12^, ^13^, ^14^, ^15^, ^16^, ^17^, ^18^, ^19^, ^20^, ^21^, ^22^, ^23^, ^24^, ^25^, ^26^, ^27^, ^28^, ^29^, ^30^, ^31^, ^32^, ^33^, ^34^, ^35^, ^36^, ^37^, ^38^, ^39^, ^40^, ^41^, ^42^, ^43^, ^44^, ^45^, ^46^, ^47^, ^48^, ^49^, ^50^ The clinical and genetic profiles of ATTR in mainland China remain elusive. Here, we report the phenotypes and genotypes of 54 patients in 39 unrelated Chinese families with ATTR.

## Materials and Methods

### Subjects and clinical data

The study was approved by the local ethics committee, and written informed consent was obtained from all patients participating in the study. We conducted a retrospective study of our cohort of 54 patients with ATTR from 17 provinces in mainland China who had been referred to our Department of Neurology, Peking University First Hospital, between January 2007 and September 2020. Clinical data and laboratory findings were collected. The severity of disability was evaluated according to the Coutinho stages of ATTR (stage 0, no symptoms; stage I, unimpaired ambulation, mostly with mild sensory, motor, and autonomic neuropathy in the lower limbs; stage II, assistance for ambulation required, mostly with a moderate motor, sensory, and autonomic impairment of the four limbs; stage III, wheelchair‐bound or bedridden status with severe sensory, motor, and autonomic involvement of all limbs).

### Mutation screening

Genetic testing was performed and *TTR* genes were analyzed. We drew 5 mL of peripheral blood and extracted genomic DNA using the method of salt precipitation. Polymerase chain reaction (PCR) primers for exons 1–4 of the *TTR* gene were designed using Primer 3 software. Standard protocols were applied for PCR. The experimental products were purified and then sequenced using the ABI 3730XL automatic sequencing machine (Applied Biosystems, USA). Detected variants were confirmed in public databases including the *Single Nucleotide Polymorphism database* (*dbSNP*) (http://www.ncbi.nlm.nih.gov/SNP/) and *Mutations in Hereditary Amyloidosis* (http://amyloidosismutations.com/cdna‐attr.html). Amino acid changes in *TTR* were numbered according to the beginning of the mature protein, as described historically, rather than including the 20 amino acid signal sequences.

### Histopathology

Histopathological analysis was conducted to obtain the direct evidence for amyloid deposits through biopsy on the tissues. Sural nerve biopsies were performed in 40 patients. Nerve specimens were split into 2 sections. The first section was fixed in 4% formaldehyde, paraffin embedded, 8µm sections, and stained with hematoxylin and eosin and Congo red. Immunohistochemistry was used to confirm that amyloid was formed by TTR (DAKO, Denmark).

The second section was fixed in 3% glutaraldehyde, post‐fixed in 1% osmium tetroxide, dehydrated through serial alcohol baths, and embedded in Epon 812 (Electron Microscopy Sciences, USA). Semithin sections of sural nerve samples for light microscopy were stained with toluidine blue. Ultrathin sections of the nerve for electron microscopy were contrasted with uranyl acetate and lead citrate.

### Reported cases in mainland China

We searched for cases in mainland China in English and Chinese that had been reported between January 2000 and September 2020. Key search terms included ‘transthyretin familial amyloid polyneuropathy’, ‘familial amyloid polyneuropathy’, ‘transthyretin amyloidosis’, ‘ATTR‐ amyloidosis’, ‘ATTR‐PN’, ‘ATTR’.

### Statistical analysis

The demographic data of the patients was included for descriptive statistics. Variables were presented as mean ± standard deviation. Continuous variables between two defined groups were compared using Student’s *t*‐test, while categorical variables were compared using Chi‐square test or Fisher’s exact test where appropriate. A level of significance of *p* < 0.05 was considered.

## Results

### Clinical presentation

A total of 54 patients (12 female, 42 male) were collected from 39 unrelated families. There were 1 case without peripheral neuropathy in Coutinho stage 0, 33 cases (61.1%) in Coutinho stage I, 10 cases (18.5%) in Coutinho stage II and III, respectively.

The average age of patients at onset (AO) was 47.8 ± 13.0 years (range 23–68 years). The late‐onset type occurred in 29 cases (53.7%) and early‐onset type in 25 cases (46.3%). The positive family history was in 22 probands (56.4%). The mean course from initial symptoms to the last visit was 4.5 ± 3.4 years (Tables 1 and 2). Three patients had a long duration of 10–20 years, with alternating diarrhea and constipation as initial symptoms.

**Table 1:** Demographic and clinical characteristics of ATTR cases with Val30Met and Ala97Ser mutations (*n* = 19).

Family/patient	Gender	Age at onset (years)	Course (years)	Coutinho stage at first visit	Mutation	Family history	Initial manifestation	Phenotype	Congo red staining in sural nerve biopsy	NCS	Duration from onset to death (years)	Cause of death
I/1	M	68	4	II	Val30Met	+	Paresthesia	PN + CTS+AN	+	Mixed	5	Respiratory failure
I/2	F	50	2	I	Val30Met	+	Paresthesia	PN + AN	+	Axonal	5	Respiratory failure
II/3	F	65	20	II	Val30Met	−	ADC	PN + C+AN	−	Mixed	25	Respiratory failure
III/4	F	67	4	I	Val30Met	+	Blurred vision	PN + C+E + Macroglossia	ND	NA	10	Sudden death
IV/5	M	64	2	I	Val30Met	−	Paresthesia	PN + AN+H + C	−	Axonal	/	/
V/6	M	43	14	I	Val30Met	+	ADC	PN + C+AN + H	−	Mixed	18	Sudden death
VI/7	M	61	1	I	Val30Met	−	Paresthesia	PN + CTS+AN	−	Axonal	/	/
VII/8	M	58	1	I	Val30Met	−	Painful paresthesia	PN + AN+C	−	Axonal	/	/
VIII/9	M	60	6	III	Val30Met	−	Paresthesia	PN + AN+C	+	Axonal	/	/
IX/10	F	60	4	I	Val30Met	−	Painful paresthesia	PN	−	NA	/	/
IX/11	M	56	4	I	Val30Met	+	Paresthesia	PN + AN	ND	NA	/	/
X/12	M	56	5	I	Val30Met	−	Painful paresthesia	PN + AN+C + Cough	−	Axonal	/	/
XI/13	M	58	5	III	Ala97Ser	+	UL Paresthesia	PN + CTS+AN + Cough	−	Axonal	10	Respiratory failure
XI/14	M	57	5	III	Ala97Ser	+	UL Paresthesia	PN + CTS+AN + H + Dysarthria + Cough	ND	Axonal	/	/
XI/15	M	64	2	I	Ala97Ser	+	UL Paresthesia	PN + AN+Cough	ND	Axonal	/	/
XI/16	M	56	8	I	Ala97Ser	+	Constipation	PN + CTS+AN + C + H + Cough	ND	Axonal	/	/
XII/17	M	56	6	III	Ala97Ser	−	Paresthesia	PN + C+AN + Dysarthria	−	Mixed	/	/
XIII/18	M	54	7	I	Ala97Ser	+	Impotence	PN + C+AN	+	Axonal	/	/
XIV/19	M	65	3	II	Ala97Ser	+	Paresthesia	PN + CTS+AN + C+ Tongue fasciculation	−	Axonal	/	/

ADC, alternating diarrhea and constipation; AN, autonomic neuropathy; Axonal, axonal neuropathy; C, cardiopathy; CTS, carpal tunnel syndrome; E, eye; H, hearing loss; K, kidney; Mixed, mixed; NA, not available; NCS, nerve conduction studies; ND, not done; PN, polyneuropathy; UL, upper limbs; “/” means no data.

**Table 2:** Demographic and clinical characteristics of ATTR cases with other mutations of study cohort (n=35).

Family/patient	Gender	Age at onset (years)	Course (years)	Coutinho stage at first visit	Mutation	Family History	Initial manifestation	Phenotype	Congo red staining in sural nerve biopsy	NCS	Duration from onset to death (years)	Cause of death
XV/20	M	28	1	I	Ala36Pro	−	Painful paresthesia	PN + AN+H	+	NA	/	/
XVI/21	M	33	1	I	Ala36Pro	+	Paresthesia	PN + CTS+AN + C	+	Axonal	/	/
XVI/22	F	30	2	II	Ala36Pro	+	Blurred vision	PN + AN+C + E	ND	Axonal	/	/
XVII/23	M	41	4	II	Glu42Gly	+	Paresthesia	PN + C+AN	−	NA	11	Sudden death
XIII/24	F	54	1	I	Glu42Gly	−	Painful paresthesia	PN + AN+C	+	Axonal	/	/
XIX/25	M	27	3	I	Glu42Gly	+	ADC	PN + AN+C + E+Cough	+	Axonal	/	/
XX/26	M	52	2	III	Phe33Leu	+	Paresthesia	PN + C+AN	−	Axonal	6	Sudden death
XX/27	M	52	1	I	Phe33Leu	+	UL Paresthesia	PN + CTS+AN + C	+	Axonal	6	Sudden death
XX/28	F	52	2	II	Phe33Leu	+	Constipation	PN + AN+C	−	Axonal	/	/
XXI/29	M	23	2	I	Phe33Val	−	Orthostatic hypotension	PN + AN	−	Axonal	4	Respiratory failure
XXII/30	M	26	2.5	I	Phe33Val	−	Impotence	PN + C+AN + E+Cough	−	Axonal	/	/
XXIII/31	M	54	10	I	Glu61Lys	−	ADC	PN + CTS+C + AN	−	Axonal	/	/
XXIV/32	M	63	4	II	Glu61Lys	−	Paresthesia	PN + C+AN	−	Axonal	/	/
XXV/33	M	28	4	I	Gly47Arg	+	Impotence	PN + C+AN	+	Axonal	/	/
XXV/34	F	27	4	I	Gly47Arg	+	Blurred vision	PN + AN+C + E	−	Mixed	/	/
XXVI/35	M	34	7	I	Glu54Gly	+	Orthostatic hypotension	PN + AN+C + E	ND	Axonal	/	/
XXVI/36	M	32	3	I	Glu54Gly	+	Painful paresthesia	PN + AN+C + E	ND	Axonal	/	/
XXVII/37	M	55	8	II	Ser77Phe	+	Diarrhea	PN + C+AN	ND	Mixed	/	/
XXVII/38	M	59	4	I	Ser77Phe	+	Paresthesia	PN + C+AN + E	−	Axonal	/	/
XXVIII/39	M	38	6	II	Lys35Asn	+	Painful paresthesia	PN + AN+C + E	+	Axonal	/	/
XXVIII/40	M	31	0.7	I	Lys35Asn	+	UL Paresthesia	PN + CTS+C	+	Axonal	/	/
XXIX/41	F	43	5	I	Val30Ala	+	Blurred vision	CTS + AN+C + E+H + K+CNS	−	Median neuropathy	/	/
XXIX/42	F	51	3	0	Val30Ala	+	Blurred vision	E + H	ND	NA	/	/
XXX/43	M	49	4	I	Val28Ser	+	Paresthesia	PN + C+AN	+	Axonal	/	/
XXX/44	M	47	8	III	Val28Ser	+	ADC	PN + C+AN	ND	NA	/	/
XXXI/45	F	44	6	III	Val30Leu	+	Paresthesia	PN + AN+C	+	Axonal	/	/
XXXI/46	M	34	10	III	Val30Leu	+	Paresthesia	PN + AN	−	NA	/	/
XXXII/47	M	42	5	I	Gly83Arg	+	Blurred vision	PN + CTS+E + AN	+	Axonal	/	/
XXXIII/48	M	60	4	II	Ser77Tyr	−	Painful paresthesia	PN + C+AN	−	Mixed	/	/
XXXIV/49	M	43	3	I	Ser50Arg	−	Paresthesia	PN + C+AN	+	Mixed	/	/
XXXV/50	M	29	7	III	Tyr114Cys	+	Diarrhea	PN + C+AN + E+CNS	ND	Axonal	/	/
XXXVI/51	M	30	1	I	Thr49Ala	+	Paresthesia	PN	−	NA	/	/
XXXVII/52	M	62	3	III	Gly47Val	−	Paresthesia	PN + CTS+C + AN	−	Mixed	/	/
XXXVIII/53	F	46	4	I	Lys35Thr	+	Paresthesia	PN + AN+C + E	ND	NA	/	/
XXXIX/54	M	45	6	I	Thr59Lys	+	Diarrhea	CTS + AN+C + E + Cough	ND	Median neuropathy	/	/

ADC, alternating diarrhea and constipation; AN, autonomic neuropathy; Axonal, axonal neuropathy; C, cardiopathy; CNS, central nervous system; CTS, carpal tunnel syndrome; E, eye; H, hearing loss; K, kidney; Mixed, mixed neuropathy; NA, not available; NCS, nerve conduction studies; ND, not done; PN, polyneuropathy; UL, upper limbs; “/” means no data.

The initial symptoms were distal paresthesia in 33 cases (61.1%), including 5 patients with paresthesia of upper limbs. Autonomic dysfunction initially occurred in 15 cases (27.8%), including gastrointestinal symptoms, sexual dysfunction and orthostatic hypotension. Blurred vision initially appeared in 6 cases (11.1%). With disease progression, all patients presented with sensorimotor or sensory neuropathy except one. Fourteen cases had carpal tunnel syndrome (CTS) (25.9%), two of whom showed isolated CTS without polyneuropathies. Of note, seventeen cases complained about obvious painful numbness (31.5%). Neurological examinations showed different proportions of sensory disturbance, muscle weakness and atrophy, and loss of deep tendon reflex (Table 3). Fourteen cases presented with ocular symptoms (25.9%), and 7 cases with hearing loss (13.0%). Forty‐nine cases (90.7%) showed autonomic dysfunction, including gastrointestinal involvement in 36 cases (66.7%), orthostatic hypotension in 26 cases (48.1%), prominent hypohidrosis in 18 cases (33.3%), sexual dysfunction in 16 cases (29.6%), and urinary dysfunction in 15 cases (27.8%). Different gastrointestinal symptoms occurred, such as alternating diarrhea and constipation (ADC) in 16 cases (29.6%), diarrhea in 12 cases (22.2%), constipation in 8 cases (14.8%), and definite upper gastrointestinal dysfunction in 7 cases (13.0%). Weight loss appeared in 28 cases (51.9%). It was worth noting that chronic cough was observed in 7 patients with Val30Met, Ala97Ser, Phe33Val, Glu42Gly mutation.

**Table 3:** Clinical, electrophysiological and pathological characteristics of symptomatic cases.

		All (*n* = 54)	Val30Met (*n* = 12)	Ala97Ser (*n* = 7)
Positive family history of probands		22 (56.4)	3(30.0)	3(75.0)
Age at onset, (mean ± SD) years		47.8 ± 13.0	59.0 ± 7.2	58.6 ± 4.2
Peripheral neuropathy		53 (98.1)	12 (100.0)	7 (100.0)
CTS		14 (25.9)	2(16.7)	4(57.1)
Neuropathic pain		17 (31.5)	5 (41.7)	4 (57.1)
Sensory impairment				
Deep		45 (83.3)	12 (100.0)	5 (71.4)
LL		44 (81.5)	11 (91.7)	5 (71.4)
UL		33 (61.1)	9 (75.0)	5 (71.4)
Superficial		51 (94.4)	12 (100.0)	7 (100.0)
LL		49 (90.7)	12 (100.0)	7 (100.0)
UL		44 (81.5)	10 (83.3)	6 (85.7)
DTR				
Only LL loss		11 (20.4)	5 (41.7)	0 (0)
Diffuse loss		39 (72.2)	7 (58.3)	7 (100.0)
Weakness				
Distal UL		41 (75.9)	10 (83.3)	6 (85.7)
Distal LL		41 (75.9)	9 (75.0)	6 (85.7)
Proximal UL		21 (38.9)	3 (25.0)	5 (71.4)
Proximal LL		19 (35.2)	3 (25.0)	5 (71.4)
Atrophy				
Distal UL		32 (59.3)	6 (50.0)	6 (85.7)
Distal LL		28 (51.9)	5 (41.7)	4 (57.1)
Proximal UL		12 (22.2)	3 (25.0)	2 (28.6)
Proximal LL		10 (18.5)	3 (25.0)	1 (14.3)
Autonomic manifestations		49 (90.7)	10 (83.3)	7 (100)
Lower cranial nerve involvement		3(5.6)	0 (0)	3 (42.9)
CNS involvement		2(3.7)	0 (0)	0 (0)
Ocular involvement		14(25.9)	1(8.3)	0 (0)
Hearing loss		7(13.0)	2(16.7)	2(28.6)
Chronic cough		7(13.0)	1(8.3)	4(57.1)
Cardiac involvement				
	Heart failure	9 (16.7)	0 (0)	2 (28.6)
	Cardiac hypertrophy with echocardiography	*n* = 44	*n* = 7	*n* = 6
		40 (90.9)	6 (85.7)	3 (50.0)
Electrophysiology		*n* = 44	*n* = 9	*n* = 7
	Axonal neuropathy	33(75.0)	6(66.7)	6 (85.7)
	Mixed neuropathy	9(20.5)	3(33.3)	1(14.3)
	Median neuropathy	2(4.5)	0 (0)	0 (0)
Positivity Congo red staining of nerve biopsy		*n* = 40	*n* = 10	*n* = 4
		16 (40.0)	3 (30.0)	3 (75.0)

Categorical variables are expressed as number (percentage); CNS, central nervous system; CTS, carpal tunnel syndrome; DTR, Deep tendon reflex; LL, lower limbs; SD, standard deviation; UL, upper limbs.

At the time of diagnosis, only 9 patients (16.7%) had symptoms of heart failure, but 40 patients (90.9%) showed cardiac hypertrophy from the echocardiography of 44 patients. Central nervous system involvement occurred in two cases, including transient ischemic attack‐like episodes, and cerebral lobe hemorrhage. Interestingly, lower cranial nerve involvement including dysarthria or tongue fasciculation occurred in three cases, all of which were Ala97Ser mutation. Other rare symptoms included one case of renal involvement and one case of macroglossia, respectively (Tables 1, 2, 3).

The differences in clinical manifestations between early‐onset and late‐onset probands from all the families were compared. A lower percentage of positive family histories and cases of Coutinho stage I, and a higher percentage of deep sensory impairment of upper limbs for late‐onset probands suggested the likelihood of more common delay of diagnosis (Table 4).

**Table 4:** Comparison of early‐onset and late‐onset ATTR probands from the study cohort (*n* = 39).

	Early‐onset (*n* = 19)	Late‐onset (*n* = 20)	*p* value
Age at onset, (mean ± SD) years	36.4 ± 8.2	59.6 ± 4.7	0.000*
Mean course, (mean ± SD) years	5.0 ± 4.3	4.5 ± 3.0	0.921
Positive family history	15 (78.9)	7 (35.0)	0.006*
Initial symptoms			
Peripheral neuropathy	10 (52.6)	15 (75.0)	0.146
Autonomic neuropathy	7 (36.8)	3 (15.0)	0.155
Others	2 (10.5)	2 (10.0)	1
Peripheral neuropathy	19 (100.0)	20 (100.0)	–
Sensory impairment			
Deep			
LL	14 (73.7)	18 (90.0)	0.235
UL	9 (47.4)	16 (80.0)	0.034*
Superficial			
LL	16 (84.2)	20 (100.0)	0.106
UL	14 (73.7)	18 (90.0)	0.235
Dissociation of deep and superficial sensation	6 (31.6)	3 (15.0)	0.273
Weakness			
Distal UL	13 (68.4)	17 (85.0)	0.273
Distal LL	12 (63.2)	17 (85.0)	0.155
Proximal UL	5 (26.3)	9 (45.0)	0.224
Proximal LL	5 (26.3)	11 (55.0)	0.069
Autonomic neuropathy	19 (100.0)	16 (80.0)	0.106
Cardiac hypertrophy with Echocardiography^1^	16 (100.0)	15 (88.2)	0.485
Coutinho stage I	15 (78.9)	9 (45.0)	0.029*

Categorical variables are expressed as number (percentage); LL, lower limbs; SD, standard deviation; UL, upper limbs.

^1^ Echocardiography was performed in 33 probands, including 16 early‐onset cases and 17 late‐onset cases.

* Statistical differences.

Forty‐four of these patients underwent electrophysiological studies at presentation. Most motor and sensory nerves showed generally axonal impairment in 33 cases (75.0%). Mixed neuropathies, including axonal and demyelinating impairment features, occurred in 9 cases(20.5%), all of which fulfilled the definite European Federation of Neurological Societies/Peripheral Nerve Society electrodiagnostic (EFNS/PNS EDX) criteria for chronic inflammatory demyelinating polyneuropathy (CIDP),^51^ including reduction of motor conduction velocity in the median nerve (4 cases), ulnar nerve (2 cases), and common peroneal nerve (3 cases), and motor distal latency prolongation in the ulnar nerve (1 case), common peroneal nerve (2 cases) and tibial nerve (4 cases). Because of CTS, two cases had isolated median neuropathy.

Totally 46 patients were followed up. Three patients received liver transplantation, five patients were treated with tafamidis, and 16 patients were treated with diflunisal. Ten patients died either from respiratory failure after being bedridden or suddenly. The median interval between onset and death was 10 years (ranging from 4 to 25). Patient 20, 36 and 37, who received liver transplantation, reported slower progression of peripheral neuropathies than before. Six years after operation, patient 20 died suddenly. The diflunisal seemed unsatisfactory to the patients, due to the continuous progression of peripheral neuropathies despite its safety. Tafamidis has just been approved in China, so we still need to follow up the efficacy and safety of this drug.

### Mutations in the *TTR* gene

A total of 22 different mutations in *TTR* gene were identified in our cohort, including Val30Met (25.6%) in 10 families, Ala97Ser in 4 families (10.3%), Glu42Gly in 3 families (7.7%), Phe33Val in 2 families (5.1%), Glu61Lys in 2 families (5.1%), and Ala36Pro in 2 families (5.1%). Each of the remaining mutations was identified in one family, including Val30Ala, Ser77Tyr, Gly47Arg, Ser50Arg, Ser77Phe, Tyr114Cys, Thr49Ala, Gly47Val, Val28Ser, Val30Leu, Gly83Arg, Phe33Leu, Glu54Gly, Lys35Asn, Lys35Thr and Thr59Lys. The mutations of Ser77Phe, Ser50Arg, Gly47Val, Glu54Gly, Thr59Lys have not been reported in mainland Chinese populations so far. Totally 9 probands with Val30Met in 10 families were of the late‐onset type (90.0%) and seven probands had negative family history during the first visit (70.0%).

**Figure 1:**
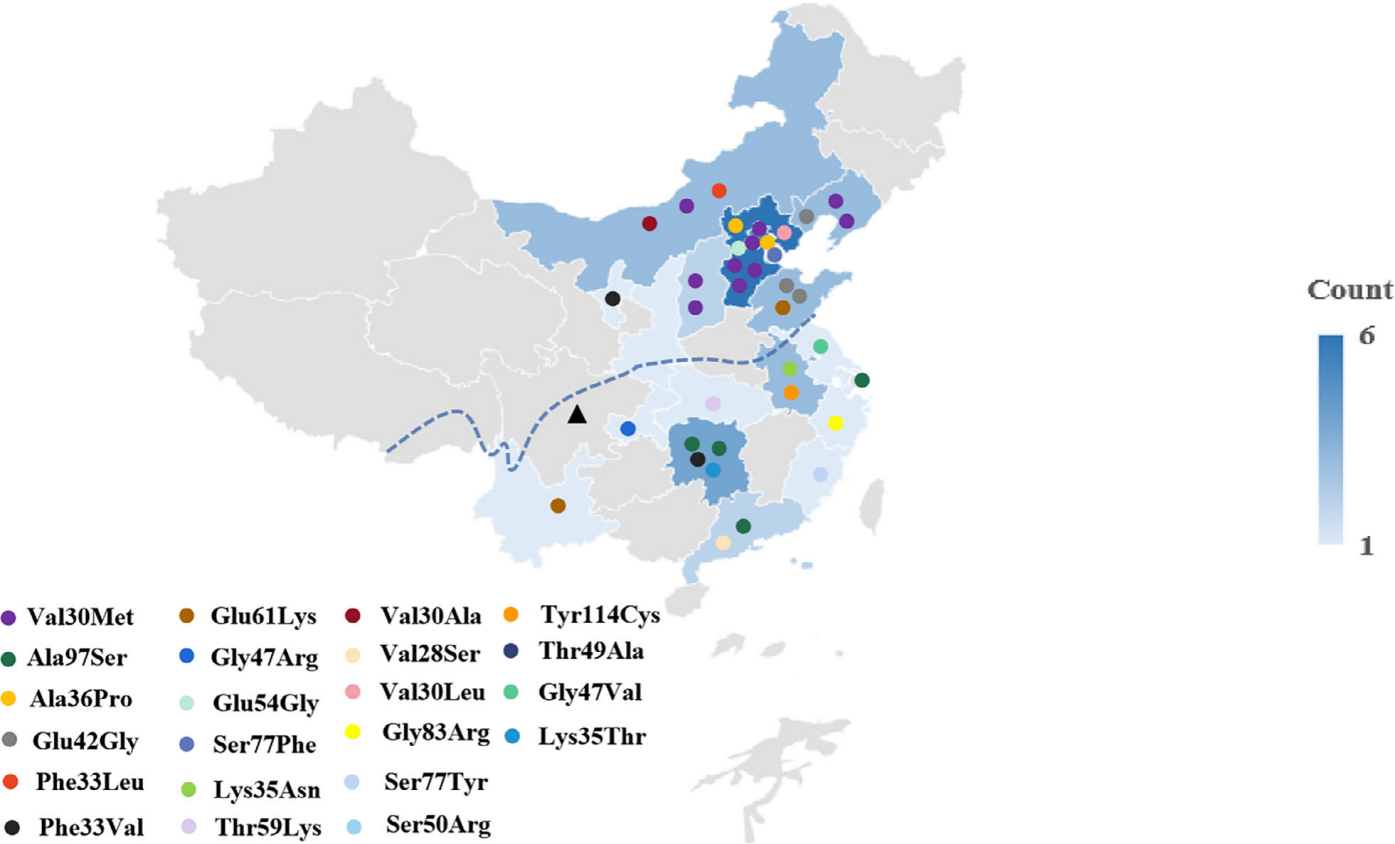
Geographical distribution of ATTR families in mainland China according to different mutations. ▲: Qinling Mountain_Huaihe River line, above the line is the north, below is the south.

Totally, 58.9% of the kindreds (23/39) originated from North China, while the remaining kindreds stemmed from South China (Fig. 1). Interestingly, all the families with Val30Met came from North China, while those with Ala97Ser came from South China. As the most common mutations in our cohort, a comparison of characteristics of cases with Val30Met and Ala97Ser was summarized in Table 3.

### Pathology

**Figure 2:**
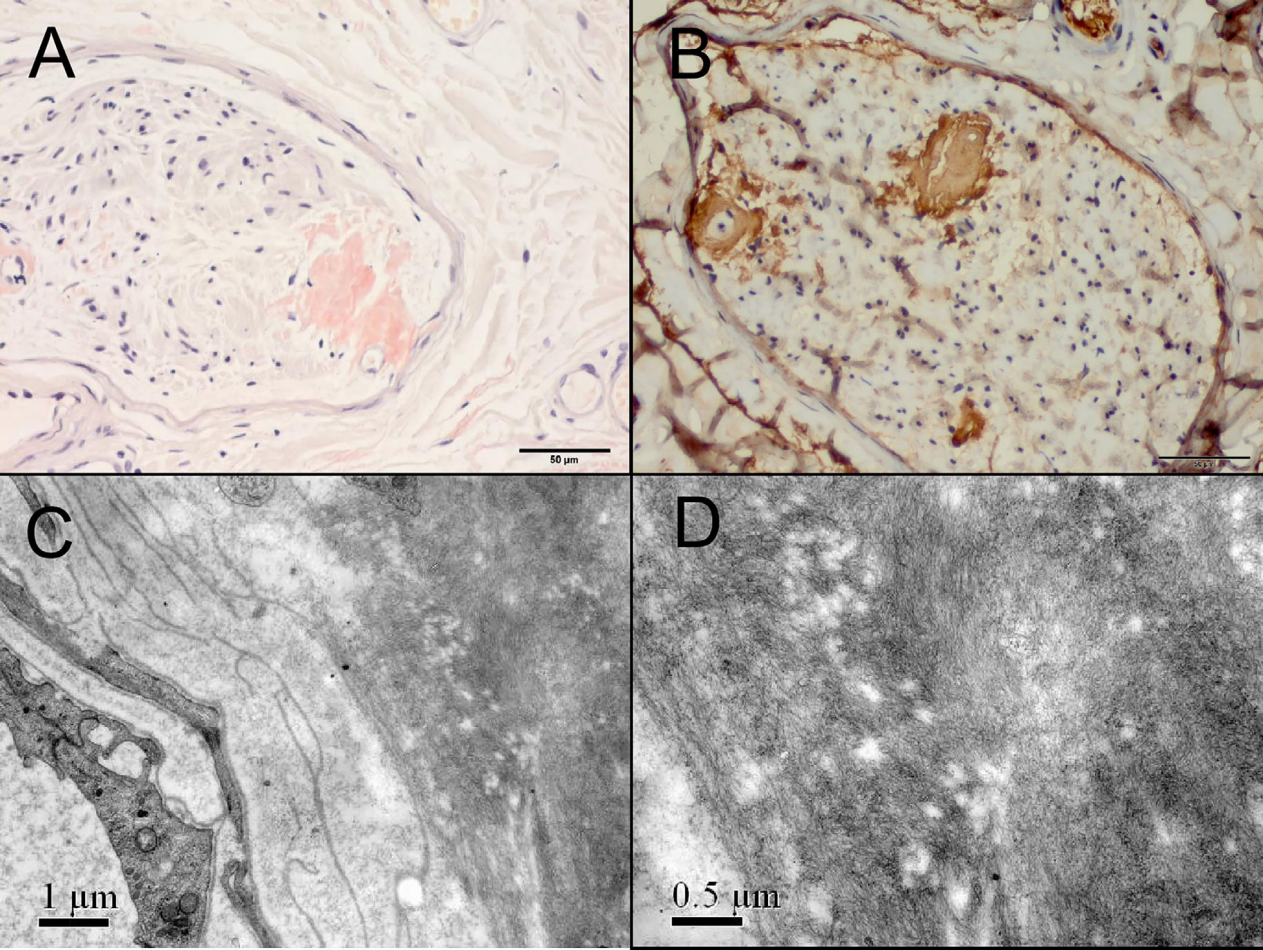
Histopathologic findings of an ATTR patient in this study. Congo red staining showed focal amyloid deposition under the epineurium, in the endoneurium and around the vessels (A), and it was positive in TTR immunohistochemistry staining (B). Ultrastructural examination further confirmed the massive filamentous amyloid deposits around the blood vessel (C, D).

Sural nerve biopsies were performed in 40 patients, all of whom presented with mild to severe axonal loss of both large and small myelinated fibers that were observed under a light microscope. Usually, clusters of regenerating myelinated fibers, degeneration of myelinated fibers, and occasionally, thin myelinated fibers and onion bulb formations were observed. Loss of nerve fibers of different diameters, especially the small myelinated and unmyelinated fibers, was further confirmed under an electron microscope. The positive rate of Congo red staining was 40.0% in sural nerves (16/40). The positive site also showed positive TTR staining. Under the electron microscope, massive filamentous amyloid deposits were observed (Fig. 2). Interestingly, the positive rate of Congo red staining was 55.6% (10/18) in early‐onset cases, and 27.3% (6/22) in late‐onset ones. It seemed that early‐onset cases had higher positive rate, though no significant difference was found (*p* = 0.069).

### Summary of reported cases of ATTR in mainland China

We reviewed 74 probands with ATTR. There were 32 kinds of mutations reported, among which Gly83Arg mutation was the dominating genotype (*n* = 11), with typical ocular involvement in all cases. Gly47Arg (*n* = 7), Val30Met (*n* = 6), and Val30Ala mutation (*n* = 6) were also frequently mentioned in previous reports, with prominent symptoms of peripheral neuropathy, autonomic dysfunction and cardiac involvement, followed by Ala97Ser mutation reported in 3 kindreds. Almost all the cases reported had prominent polyneuropathy and autonomic neuropathy, except some special cases that were closely related to a special genotype. Patients with Gly83Arg, Lys35Thr or Arg34Gly mutations mainly manifested themselves as ocular symptoms, and these cases were usually reported by ophthalmologists for presenting with blurred vision initially. Other special symptoms included chronic dry cough in Val30Ala and Ala97Ser, prominent psychiatric symptoms in Asp18Gly and Leu12Pro, prominent cardiac symptoms in His88Arg, and renal involvement in Leu55Pro (Table 5). The AO ranged from 17 to 69 years old.

**Table 5:** Clinical presentation and genetic phenotype of reported ATTR cases in mainland China.

Number	Mutation	Reported kindreds	Associated clinical features	References
1	Gly83Arg	11	E(main), PN, C	21, 22, 27, 34, 40, 42, 45, 48
2	Gly47Arg	7	PN, AN, CNS, C	14, 44
3	Val30Met	6	PN, AN, C, K, E	14, 38, 41, 44, 46
4	Val30Ala^α^	6	PN, AN, C, H, LM	12, 13, 14, 33, 36, 41, 44
5	Ala97Ser^α^	3	PN, AN, C	11, 18, 19
6	Glu54Lys	3	PN, AN, C, E	14, 32, 44, 49
7	Val30Leu	3	PN, AN, C, E	17, 30, 43
8	Ala36Pro	3	PN, AN, E, C	15, 26, 41
9	Gly47Glu	2	PN, AN, C	14, 44
10	Asp38Val	2	PN, AN, C	14, 44
11	Gly53Glu	2	PN, C	14, 44
12	Lys35Asn	2	PN, AN, C	14, 44
13	Lys35Thr	2	E(main), PN, AN	31, 37
14	Leu55Arg	2	PN, AN, C, E	15, 31
15	Tyr114Cys	2	PN, AN, C, E, K	14, 28
16	Phe33Leu	2	PN, AN, C	14, 41, 44
17	Thr49Ala	1	PN, AN, C, E	15
18	Asp18Gly	1	PN, LM	25
19	Glu54Gln	1	PN, AN, C	14, 44
20	Glu61Lys	1	PN, AN, C	14
21	Phe64Ser	1	PN, AN, C, LM, E	23
22	His88Arg	1	C	14
23	Ile107Met	1	PN, AN, E	20
24	Leu12Pro	1	AN, C, H, LM, E	50
25	Arg34Gly	1	E	35
26	Ser77Tyr	1	PN, AN, C	14
27	Val28Ser	1	PN, AN, C, E	47
28	Leu55Pr	1	PN, AN, C, K	39
29	Tyr116Ser	1	PN, AN, C, E	15
30	Tyr69His	1	PN, AN, C, CNS	14
31	Glu42Gly	1	PN, AN, C	41
32	Phe33Val	1	PN, AN, E	41

AN, autonomic neuropathy; C, cardiopathy; CNS, central nervous system; E, eye; H, hearing loss; K, kidney; LM, leptomeningeal; PN, polyneuropathy; α, Chronic dry cough as a special symptom appeared in one case with these two mutations, respectively.

## Discussion

In our study, we reported the clinical and genetic profiles of ATTR in 54 patients from 39 families, which is currently the largest ATTR cohort in mainland China. A study from Taiwan investigated the clinical features and mutational spectrum of a cohort of 79 patients with 6 kinds of *TTR* mutations from 57 pedigrees, which is the largest ATTR cohort in Chinese populations so far. It was found that Ala97Ser mutation was the most common cause of ATTR in Taiwan, accounting for 91.2% of the pedigrees.^9^ In our cohort, patients with 22 kinds of different *TTR* mutations came from 17 provinces across mainland China, reflecting the more real genotypes of Chinese populations.

In our cohort, *TTR* Val30Met mutation was the most common cause of ATTR in mainland China, especially in North China, accounting for 25.6% of the families. Only 30.0% of the probands had the positive family history, and late‐onset type occurred in all the patients except one, who showed simple alternating diarrhea and constipation at the age of 43, more than 10 years earlier than other systems involvement. That is different from the endemic area in Portugal and Japan, where early‐onset type and known family history are common.^52^, ^53^ The fact that very few *TTR* Val30Met mutation cases have been reported before in mainland China probably means that such cases might have been greatly underestimated. As in the Taiwan cohort, our patients with the *TTR* Ala97Ser mutation usually presented with a late‐onset peripheral neuropathy and autonomic dysfunction.^9^ Unlike the genetic situation in the north of China, *TTR* Ala97Ser mutation is the dominant mutation type in the south. According to previous studies, the most common mutation is Thr60Ala in the UK and US, Val30Met in Japanese non‐endemic areas, and Asp38Ala in South Korea, indicating different genetic characteristics of ATTR in non‐endemic areas of the world.^54^, ^55^, ^56^, ^57^ Additionally, another 20 kinds of *TTR* mutations have been found in our cohort, five of which have never been reported in mainland China before. It has to be acknowledged that our center is in Beijing, a northern city of China, which may lead to genotype bias, indicating the need for multicenter epidemiological investigations of ATTR in the future.

In the cohort, the most common clinical manifestations were peripheral neuropathy, in which, chronic sensory or sensorimotor polyneuropathy was the dominant type. Above 30% of the cases had neuropathic pain, which could occur as the initial symptoms, indicating early small nerve fiber involvement in ATTR.^9^ Generally speaking, autonomic neuropathy was more common in early‐onset patients in previous reports.^53^ From our experience, autonomic neuropathy in late‐onset patients was not few, but showed a mild degree, which tended to be neglected. That is an important cause of delayed diagnosis, if autonomic neuropathy is considered the distinctive feature of ATTR.^53^ Cardiomyopathy, although frequently observed in this study, was not an onset symptom, which was consistent with the results of a previous study in Chinese populations.^14^ Cardiac involvement was usually associated with symptoms of heart failure, and indicated the clinical course of the disease.^14^, ^52^ We observed no more than nine patients with symptoms of heart failure during diagnosis, but over 90% of the patients had abnormal findings in echocardiography, underscoring the importance of cardiac evaluation of each patient with ATTR, even among those without heart symptoms. Ocular amyloidosis occurred in 25.9% of our patients, which was the case with Mayo Clinic patients with ATTR (24%).^58^ The present study also indicated that hearing loss was not a rare symptom, suggesting amyloid deposition in the various anatomical structures of the inner and middle ear.^59^

Renal involvement, including a nephritic syndrome and progressive renal failure, which occurred in about one in three patients in Portugal,^53^ was rare in our cohort. Central nervous system involvement was observed in one case with the Tyr114Cys, who presented with transient ischemic attack‐like episodes of dysarthria, hemihypoesthesia, hemiplegia and psychiatric symptoms, and in another case with Val30Ala as evidenced by cerebral lobe hemorrhage, thus highlighting the need for early investigations of central nervous system amyloidosis.^60^ Interestingly, chronic cough was observed in 7 patients with *TTR* Val30Met, Ala97Ser, Phe33Val, Glu42Gly mutation. Nevertheless, the cause of chronic dry cough remains unknown, but could be attributed to deposition of amyloid in the vagal nerve or denervation hypersensitivity of the upper airways.^19^ Three patients had lower cranial nerve involvement, including dysarthria or tongue fasciculation with Ala97Ser mutation, to our knowledge, which was not mentioned in previous studies with Ala97Ser mutation.^9^, ^10^

In our cohort, we confirmed that 75.0% of the cases had sensory‐motor axonal neuropathies in the electrophysiological studies, while 20.5% of the cases showed mixed neuropathies with demyelinating patterns, which made it difficult to differentiate ATTR from some acquired demyelinating neuropathies.^61^ Isolated CTS has been found in our cohort, which could be an early neuropathy pattern of ATTR.^4^, ^5^ Sural biopsy was less likely to detect amyloid deposition, but several other biopsy sites other than nerves may contribute more to the diagnosis of ATTR.^62^ Interestingly, the trend that the positive rate of amyloid deposition of sural nerves was lower in late‐onset patients was found, possibly because the blood‐nerve barrier in the distal part of peripheral nerves may be preserved later in late‐onset ATTR.^63^ We found mild to severe loss of myelinated fibers of different diameters, especially the small‐diameter and unmyelinated fibers, which started from the asymptomatic stage of ATTR.^64^ Thin myelinated fibers and onion bulb formations were observed occasionally, which indicated involvement of myelin. Similar pathologic findings were observed in previous studies, which revealed severe axonal loss and occasional segmental demyelination‐remyelination in nerve biopsy.^3^

We have reported the largest ATTR cohort in mainland China. There are several limitations to our study. Firstly, our study is a retrospective study, in which recall bias may affect the results. Secondly, in our cohort, more cases come from North China, so the clinical and genetic characteristics may not reflect the real feathers of mainland China. Finally, all the probands were admitted to our Neurological Department first, so in our cohort, it was difficult to include ATTR cases without peripheral neuropathy. Therefore, future multicenter or multi‐disciplinary studies are needed to identify ATTR phenotypes and genotypes in mainland China.

## Conclusion

This study reported the clinical and genetic characteristics of ATTR in a single center in mainland China, and revealed the heterogeneity of ATTR phenotypes and genotypes in Chinese populations. Val30Met is the most common mutation type in mainland China, but it might be greatly underestimated because of the high proportion of sporadic and late‐onset patients. There are genotypic differences between North and South China.

## Conflict of Interest

The authors declare no conflict of interest.
